# Entropy Production Analysis of a Vertical Mixed-Flow Pump Device with Different Guide Vane Meridians

**DOI:** 10.3390/e24101370

**Published:** 2022-09-27

**Authors:** Yanjun Li, Yi Zhong, Fan Meng, Yunhao Zheng, Danghang Sun

**Affiliations:** 1 Research Center of Fluid Machinery Engineering and Technology, Jiangsu University, Zhenjiang 212013, China; 2 Wenling Fluid Machinery Technology Institute, Jiangsu University, Wenling 317525, China

**Keywords:** mixed-flow pump device, guide vane meridians, entropy production, numerical simulation

## Abstract

With the aim of investigating the influence of guide vane meridians on the external characteristics and internal flow field of the mixed-flow pump device, this research constructed seven guide vane meridians and applied computational fluid dynamic (CFD) and entropy production theory to investigate the spread of hydraulic loss in a mixed-flow pump. As observed, when the guide vane outlet diameter *D*_gvo_ decreased from 350 mm to 275 mm, the head and efficiency increased by 2.78% and 3.05% at 0.7 *Q*_des_, respectively. At 1.3 *Q*_des_, when *D*_gvo_ increased from 350 mm to 425 mm, the head and efficiency increased by 4.49% and 3.71%, respectively. At 0.7 *Q*_des_ and 1.0 *Q*_des_, the entropy production of the guide vane increased with the increase of *D*_gvo_ due to flow separation. When *D*_gvo_ < 350 mm, at 1.0 *Q*_des_ and 1.3 *Q*_des_, entropy production of the outlet channel increased as *D*_gvo_ decreased owing to the excessive flow rate, but at 0.7 *Q*_des_, entropy production did not change much. When *D*_gvo_ > 350 mm, at 0.7 *Q*_des_ and 1.0 *Q*_des_, due to the expansion of the channel section, the flow separation intensified, which resulted in an increase of the entropy production, but the entropy production decreased slightly at 1.3 *Q*_des_. These results provide guidance for improving the efficiency of pumping stations.

## 1. Introduction

A vertical mixed-flow pump device with a large flow and high efficiency has been widely used in industrial manufacturing [1] and agricultural irrigation [2]. When the water flows from the impeller of the mixed-flow pump, some vortex energy is generated, most of which can be converted into pressure energy by a guide vane. However, the hydraulic interference effect of the outlet channel on the outflow of the guide vane was neglected when the mixed-flow pump was designed. Therefore, the optimal effluent cyclone may not be achieved by the conventional hydraulic design. Thus, by changing the guide vane meridians, the optimal swirl distribution under the hydraulic interference of the guide vane-outlet channel can be explored.

However, due to limitations of the experimental tools, it is difficult to fabricate a mixed-flow pump device with different guide vane meridians. In recent years, computational fluid dynamics (CFD) has developed rapidly. It has been widely used in numerical simulation of the internal flow of mixed-flow pumps [3,4,5,6,7,8,9,10,11]. Research on the mixed-flow pump device based on CFD can be divided into two main categories. One focuses on the impeller-guide vane dynamic interference effect [12,13,14,15]. For example, Fu [16] carried out numerical simulation on the mixed-flow pump with 12 matching schemes with different amounts of guide vane blades and impeller blades. The results showed that when the impeller blade number was fixed, the increase in the guide vane blade number resulted in a better matching between the guide vane and impeller, improving the efficiency and head of the mixed-flow pump. Li et al. [17] studied the pressure pulsation of mixed-flow pumps with different guide vane thicknesses and found that a thinner guide vane leads to worse matching. The amplitude of pressure pulsation in the middle of the guide vane was increased by 7.8%. Zhang et al. [18] changed the guide vane hub radius of the mixed-flow pump while keeping the shape of blade. The results showed that under the designed flow conditions, when the hub radius of the guide vane was increased by 11 mm, the guide vane could more effectively recover the residual circulation at the outlet of the impeller, and the efficiency was improved by 2.3%, compared with the initial scheme. Zhu [19] used the global dynamic criterion algorithm to optimize 18 parameters, such as the outlet angle, inlet angle, and wrap angle of the impeller and guide vane, to make the guide vane inlet angle match the impeller outlet angle better, resulting in a more stable flow field in the impeller-guide vane interaction area. Under the designed flow conditions, the efficiency was increased by 1.94%. The other CFD-based mixed-flow pump device studies have centered on the hydraulic coherence between the guide vane and the outlet channel. Zheng et al. [20] studied the distribution characteristics of the hydraulic loss of mixed-flow pump devices under different inflow angles. The results showed that with the increase of the inlet angle, the interference effect between the outlet channel and guide vane was weakened, which resulted in an increase of hydraulic loss in the outlet channel. Pei et al. [21] analyzed the flow characteristics of low-head pumps with different impeller-guide vane distances, and they found that increasing the distance between the guide vane and the impeller affected the hydraulic interference between the guide vane and the outlet channel. Under the small flow rate condition, as the impeller-guide vane distance increased, the hydraulic loss inside the outlet channel increased, and it decreased under the condition of a small flow rate.

However, only the total hydraulic loss in the components can be calculated by the traditional method, and it is difficult to obtain the spread of hydraulic loss accurately. Entropy production theory can not only quantify the hydraulic loss, but also obtain the loss specific location distribution. Kock F. [22] and Herwig H. [23] introduced the Reynolds time-averaged equation into entropy production theory and made it possible to obtain entropy production in CFD post-processing. In recent years, some researchers, within the framework of entropy production theory, studied the internal flow of mixed-flow pumps. Zhang et al. [24] used entropy production theory to analyze the spatial distribution of hydraulic loss of mixed-flow pumps with different pressure chambers. They found that when the flow rate was 800–1200 m^3^/h, the entropy production value of the mixed-flow pump with the guide vane pressure chamber was higher than that with the volute pressure chamber. However, when the flow rate was 1200–1600 m^3^/h, it was lower. The energy loss of the mixed-flow pump was studied within the framework of entropy production theory by Ji et al. [25]. Their study showed that when the tip clearance was increased from 0.2 mm to 1.1 mm, the entropy production of the pump was increased by 142%, and the entropy production of the guide vane was decreased by 21.8%. Cao et al. [26] analyzed the hydraulic loss distribution characteristics of the annular volute mixed-flow pump based on entropy production theory, and they found that the entropy production in the annular volute was much larger than that of other parts. Combined with internal flow field analysis, it was concluded that the entropy production at this position was caused by flow impact and separation. These studies showed that entropy production theory is a reliable prediction method, which could provide a reference for the optimal design of mixed-flow pumps.

Few studies have centered on the influence of different guide vane meridians on the spatial distribution of hydraulic loss in mixed-flow pumps. This study carried out an unsteady numerical simulation on seven mixed-flow pump devices with different guide vane meridians. A comparison with the experimental data was made to verify the reliability of the numerical simulation results. Meanwhile, entropy production theory was introduced to evaluate the distribution of hydraulic loss.

## 2. Numerical Simulation

### 2.1. Three-Dimensional Model

**Figure 1:**
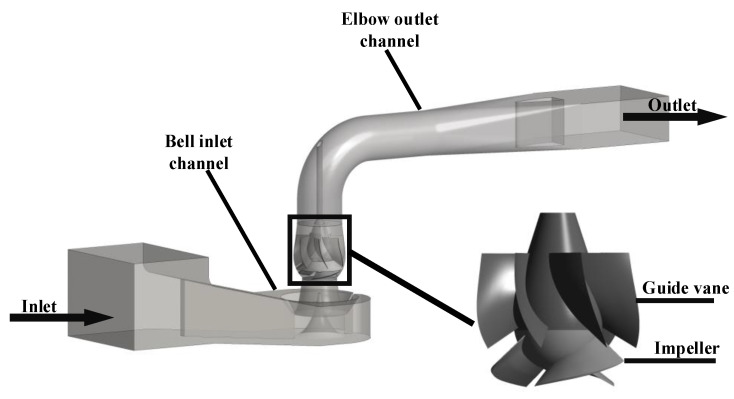
Three-dimensional model of the mixed-flow pump device.

Figure 1 shows the three-dimensional model of the mixed-flow pump device, including the bell inlet channel, elbow outlet channel, guide vanes, and impeller. The design flow rate *Q*_des_ was 336 L/s, the rated rotation speed *n* was 1218.8 r/min. The diameter of the impeller was 320 mm, the number of impeller blades *Z*_1_ was 3, and the tip clearance of the impeller was 0.15 mm.

**Figure 2:**
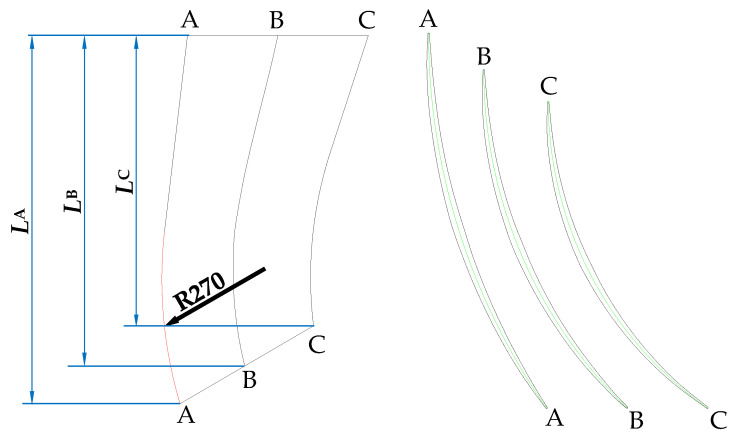
Meridional shape of the guide vane blade (A-A, B-B and C-C: section of guide vane blade at shroud side, middle and hub side; *L*_A_, *L*_B_ and *L*_C_: length of guide vane at section A-A, B-B and C-C).

**Figure 3:**
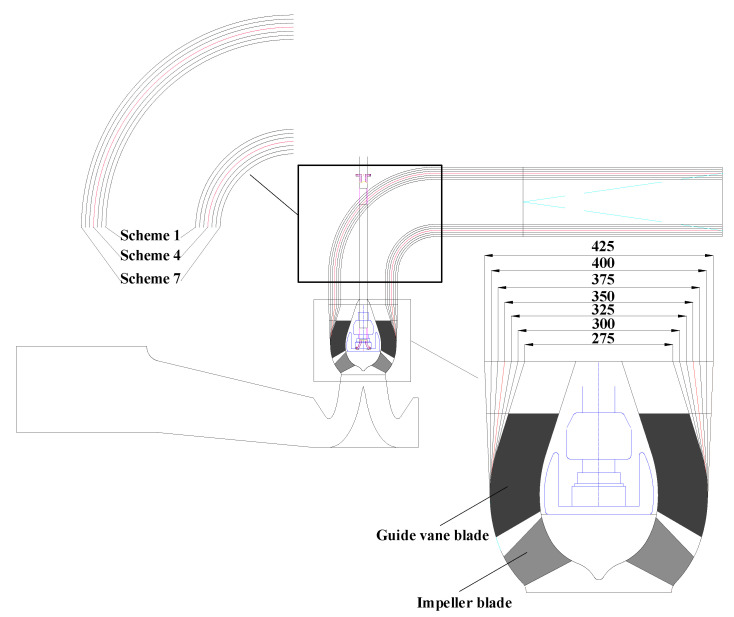
Mixed-flow pump device with various guide vane meridians.

In this paper, only the meridian profile of the guide vane and the outlet channel were changed. Figure 2 shows the meridional shape of the guide vane blade and Table 1 shows the specific parameters of the blade. The shroud profile of the guide vane consisted of an arc with a radius of 270 mm and a straight line. With the section A-A, B-B, and C-C unchanged, changing the slope of the straight line led to variable diameters of the guide vane outlet *D*_gvo_, which were 275, 300, 325, 350, 375, 400, and 425 mm. The initial size was 350 mm. The guide vane was modelled by CF turbo. In order to ensure that the outlet channel was smoothly connected with the guide vane, seven schemes were also set for the outlet channel, as shown in Figure 3.

### 2.2. Mesh Generation

**Figure 4:**
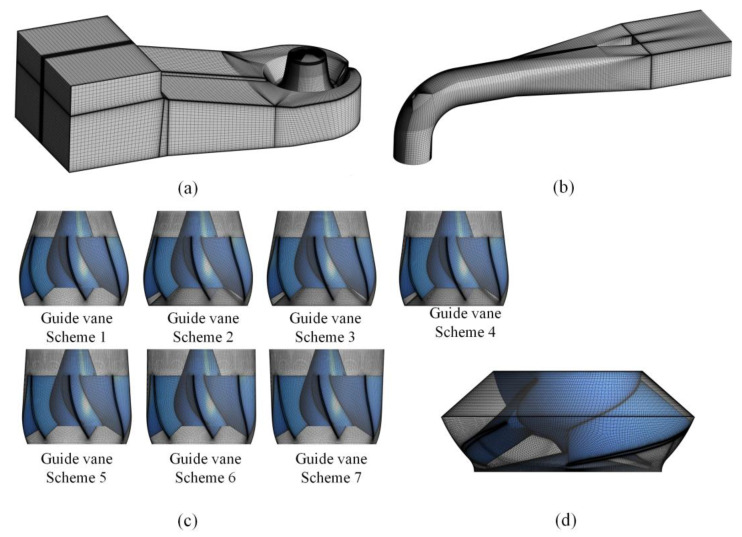
Mesh of the computation domain: (**a**) inlet channel; (**b**) outlet channel; (**c**) guide vane with various meridians; and (**d**) impeller.

With the aim of ensuring the accuracy of the calculation, all components were divided by structural mesh, as shown in Figure 4. UG was employed to model the inlet channel and outlet channel, ICEM was used to divide the structural grid, and Turbo Grid was employed to generate the structural mesh of the guide vane and impeller.

### 2.3. Mesh Independence Analysis

With the purpose of minimizing the effect of the mesh number on the simulation accuracy, the transient simulation of the mixed-flow pump device with 350 mm *D*_gvo_ was carried out. Table 2 shows the head and efficiency of models with a different number of grid nodes. When the number of grid nodes was greater than 4.98 million, the changes of the head and efficiency were within 0.24% and 0.14%, respectively. The grid number of the mixed-flow pump models of the seven schemes was between 8.72 million and 9.22 million, which can be used to make sure the simulation results are accurate. Furthermore, the maximum value of y+ under the seven schemes is less than 90.

### 2.4. Control Equations and Boundary Conditions

Based on the computational fluid dynamics software CFX2020, the Reynolds time-averaged equation was employed to execute the steady simulation of mixed-flow pump devices with different guide vane meridians. With the steady simulation results as the initial data, the transient simulation was carried out. The boundary conditions are shown in Table 3. The time step was 4.1 × 10^−4^ s, that is, the time for the impeller to rotate by 3°, and the total time was 4.92287 × 10^−1^ s, that is, the time required for the impeller to rotate 10 turns. Since the shape of all walls remains unchanged during the simulation, no slip is set for all solid walls [27].

### 2.5. Entropy Production Theory

Based on the second law of thermodynamics, the thermodynamic process is always accompanied by entropy production, which is mainly caused by heat transfer and dissipation. Since the specific heat capacity of water is large, the temperature can be considered constant during the operation of the pump device, so the entropy production in the pump device is only determined by dissipation. Dissipation is mainly composed of three parts: the direct dissipation ${\dot{S}}_{\bar{\mathrm{pro}}}^{\prime \prime \prime }$ can be calculated by Equation (1) [20]; the indirect dissipation ${\dot{S}}_{{\mathrm{pro}}^{\prime }}^{\prime \prime \prime }$ can be calculated by Equation (2) [20]; and the wall dissipation ${\dot{S}}_{\mathrm{pro},w}^{\prime \prime }$ can be calculated by Equation (3) [21]:

(1) $$\begin{array}{c} \begin{array}{c} {\dot{S}}_{\bar{\mathrm{pro}}}^{\prime \prime \prime }=\frac{\mu }{T}\left[{\left(\frac{\partial \bar{u_{x}}}{\partial y}+\frac{\partial \bar{u_{y}}}{\partial x}\right)}^{2}+{\left(\frac{\partial \bar{u_{x}}}{\partial z}+\frac{\partial \bar{u_{z}}}{\partial x}\right)}^{2}+{\left(\frac{\partial \bar{u_{z}}}{\partial y}+\frac{\partial \bar{u_{y}}}{\partial z}\right)}^{2}\right]\\ \;+\frac{2\mu }{T}\left[{\left(\frac{\partial \bar{u_{x}}}{\partial x}\right)}^{2}+{\left(\frac{\partial \bar{u_{y}}}{\partial y}\right)}^{2}+{\left(\frac{\partial \bar{u_{z}}}{\partial z}\right)}^{2}\right] \end{array} \end{array}$$

(2) $${\dot{S}}_{{\mathrm{pro}}^{\prime }}^{\prime \prime \prime }=\frac{\mu }{T}\left[\bar{{\left(\frac{\partial u_{x}{}^{\prime }}{\partial y}+\frac{\partial u_{y}\prime }{\partial x}\right)}^{2}}+\bar{{\left(\frac{\partial u_{x}{}^{\prime }}{\partial z}+\frac{\partial u_{z}{}^{\prime }}{\partial x}\right)}^{2}}+\bar{{\left(\frac{\partial u_{z}{}^{\prime }}{\partial y}+\frac{\partial u_{y}\prime }{\partial z}\right)}^{2}}\right]+\frac{2\mu }{T}\left[\bar{{\left(\frac{\partial u_{x}{}^{\prime }}{\partial x}\right)}^{2}}+\bar{{\left(\frac{\partial u_{y}\prime }{\partial y}\right)}^{2}}+\bar{{\left(\frac{\partial u_{z}{}^{\prime }}{\partial z}\right)}^{2}}\right]$$

(3) $${\dot{S}}_{\mathrm{pro},w}^{\prime \prime }=\frac{\tau \cdot v}{T}$$

where $\bar{u_{x}}$, $\bar{u_{y}}$, $\bar{u_{z}}$ represents the time-averaged velocities in the *x, y, z* directions, respectively; $u_{x}{}^{\prime }$, $u_{y}\prime$, $u_{z}{}^{\prime }$ stand for fluctuating velocities in the *x, y, z* directions, respectively; T represents temperature and μ represents fluid viscosity.

Since the fluctuating velocities $u_{x}{}^{\prime }$, $u_{y}\prime$, $u_{z}{}^{\prime }$ cannot be obtained by the calculation results, in the *SST k-ω* turbulence model, ${\dot{S}}_{{\mathrm{pro}}^{\prime }}^{\prime \prime \prime }$ can be calculated by Equation (4) [21]:

(4) $${\dot{S}}_{{\mathrm{pro}}^{\prime }}^{\prime \prime \prime }=\frac{\rho k}{T}$$

where ρ is the fluid density and *k* is turbulent eddy dissipation.

Entropy production is defined by the integral entropy production rate:

(5) $$S_{\bar{\mathrm{pro}}}=\int_{V}{\dot{S}}_{\bar{\mathrm{pro}}}^{\prime \prime \prime }dV$$

(6) $$S_{{\mathrm{pro}}^{\prime }}=\int_{V}{\dot{S}}_{\mathrm{pro}\prime }^{\prime \prime \prime }dV$$

(7) $$S_{\mathrm{pro},w}=\int_{s}{\dot{S}}_{\mathrm{pro},w}^{\prime \prime }ds$$

(8) $$S_{\mathrm{pro}}=S_{\bar{\mathrm{pro}}}+S_{{\mathrm{pro}}^{\prime }}+S_{\mathrm{pro},w}$$

where $S_{\bar{\mathrm{pro}}}$ indicates direct entropy production; $S_{{\mathrm{pro}}^{\prime }}$ indicates indirect entropy production; $S_{\mathrm{pro},w}$ stand for wall entropy production; and $S_{\mathrm{pro}}$ stand for total entropy production.

## 3. Verification of Simulation

**Figure 5:**
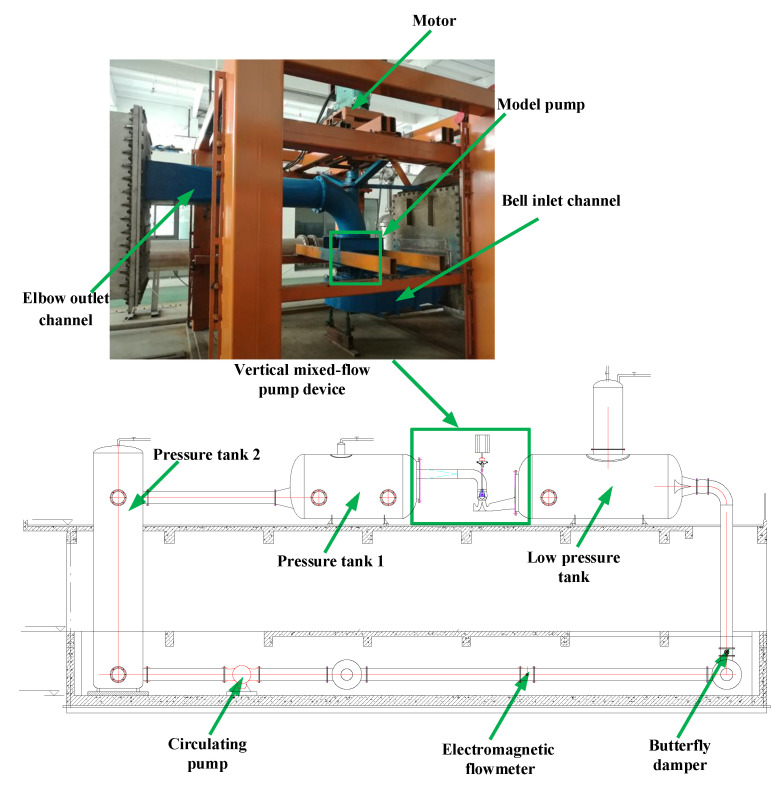
Experimental system.

In this paper, the energy characteristic curve of the pump device model was measured by a four-quadrant multi-function test rig of hydraulic machinery. The test bench adopted a vertical structure, as presented in Figure 5. A flowmeter with measurement uncertainty *E*_Q_ of ±0.2% was employed to measure the flow rate, and the measurement uncertainty of the head detector *E*_H_ was ±0.1%. In the range of 1–500 Nm, the uncertainty of the torque measurement *E*_M_ was ±0.1% and the uncertainty of the rotating speed measurement *E*_n_ was ±0.1%. The systematic uncertainty of the test bench *E*_S_ consisted of the head, shaft power, and flow measurement uncertainty, which can be calculated by Equation (9):

(9) $$E_{S}=\sqrt{E_{H}^{2}+E_{T}^{2}+E_{Q}^{2}}=0.26\%$$

Ten repeated measurements were carried out at the highest efficiency point, and the random uncertainty of the efficiency test *E*_R_ was calculated to be ±0.13%. Therefore, the efficiency test comprehensive uncertainty *E*_η_ could be calculated by Equation (10):

(10) $$E_{\eta }=\sqrt{E_{S}^{2}+E_{r}^{2}}=0.291\%$$

**Figure 6:**
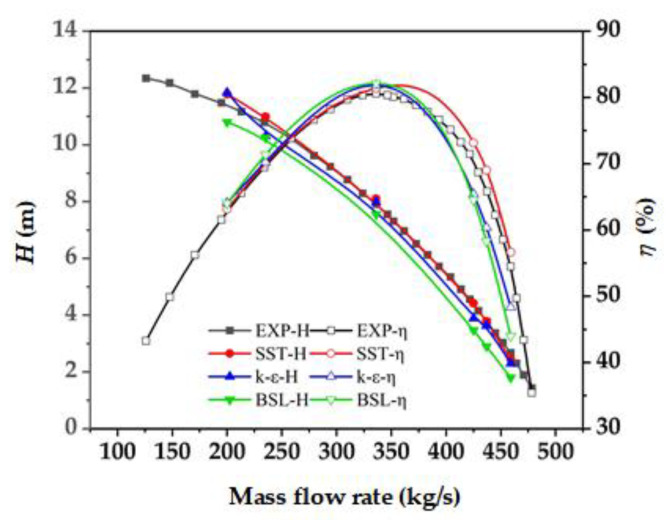
Comparison of simulation and experiment results.

In Figure 6, a comparison is made between the values of the experiment and simulation in different turbulence models. As Figure 6 shows, the simulation value in the SST turbulence model is closer to the experimental value, while the simulation value in k-ε and BSL turbulence models is significantly smaller than the experimental value at large flow rate condition. Therefore, in this paper, the SST turbulence model is used to simulate the mixed-flow pump device. For simulation results in SST turbulence simulation, under the design flow, the relative error between the simulated head and the experimental value was less than 2.5%, and that between the simulated efficiency and the experimental value was less than 1%. The maximum relative error under other conditions was less than 6%. This indicates that the consistency between the numerical simulation value and the experimental measurement value met the requirements, and the numerical simulation results are reliable.

## 4. Results and Discussion

### 4.1. Comparison between Hydraulic Loss and Entropy Production

**Figure 7:**
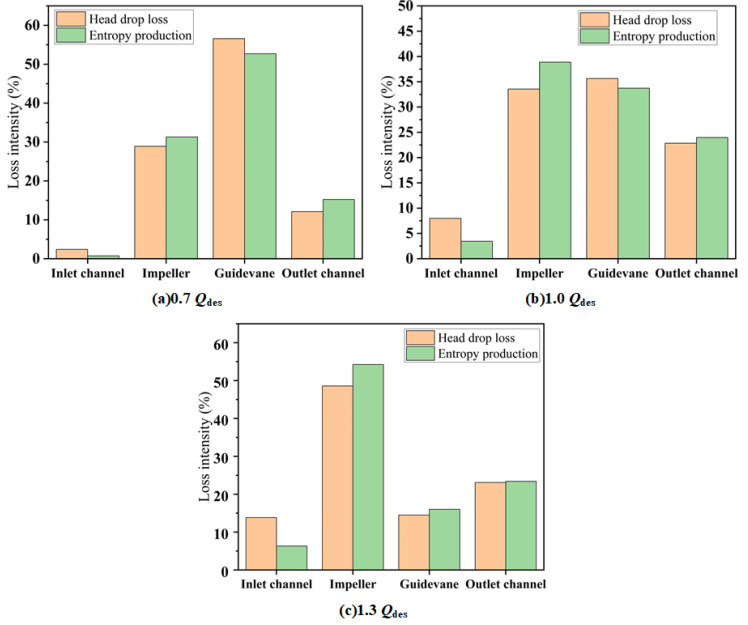
Comparison of hydraulic loss and entropy production for each component at various flow rate conditions: (**a**) 0.7 *Q*_des_; (**b**) 1.0 *Q*_des_; and (**c**) 1.3 *Q*_des_.

This paper, within the framework of entropy production theory, analyzed the energy characteristics of the mixed-flow pump device. Therefore, it is necessary to confirm that the entropy production theory can indeed be used to characterize the hydraulic loss. Figure 7 shows a comparison between the ratio of hydraulic loss to total loss and the ratio of entropy production to total production of each component, with a mixed-flow pump of the initial scheme under different flow conditions. Under different flow rates, the hydraulic loss of each component showed the same trend as entropy production, which proves that entropy production theory is a reliable method to analyze hydraulic loss.

### 4.2. External Characteristics of Mixed-Flow Pump Devices with Different Guide Vane Meridians

**Figure 8:**
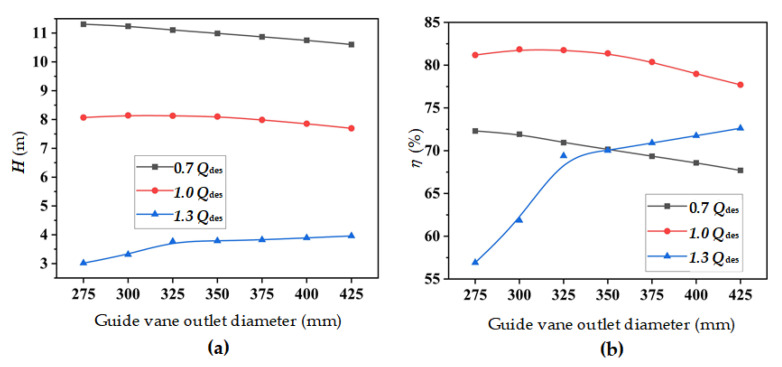
External characteristics of a mixed-flow pump device with different guide vane outlet diameters: (**a**) head; and (**b**) efficiency.

Figure 8 depicts the external characteristic of a mixed-flow pump device with different guide vane meridians under various flow rate conditions. Under 0.7 *Q*_des_, as the *D*_gvo_ decreased, the efficiency and head of the mixed-flow pump device increased. When *D*_gvo_ decreased from 350 mm to 275 mm, the efficiency and head increased by 3.05% and 2.78%, respectively. At 1.0 *Q*_des_, when *D*_gvo_ was in the range of 300 mm to 350 mm, the efficiency and head of the mixed-flow pump device hardly changed, but when *D*_gvo_ < 300 mm or *D*_gvo_ > 350 mm, the efficiency and head showed a distinct downward trend. At 1.3 *Q*_des_, when *D*_gvo_ < 350 mm, the efficiency and head of the pump device increased along with the increase of diameter. When *D*_gvo_ increased to 425 mm, compared with the original scheme, the efficiency and head increased by 3.71% and 4.49%, respectively.

### 4.3. Entropy Production of Mixed-Flow Pump Devices with Different Guide Vane Meridians

**Figure 9:**
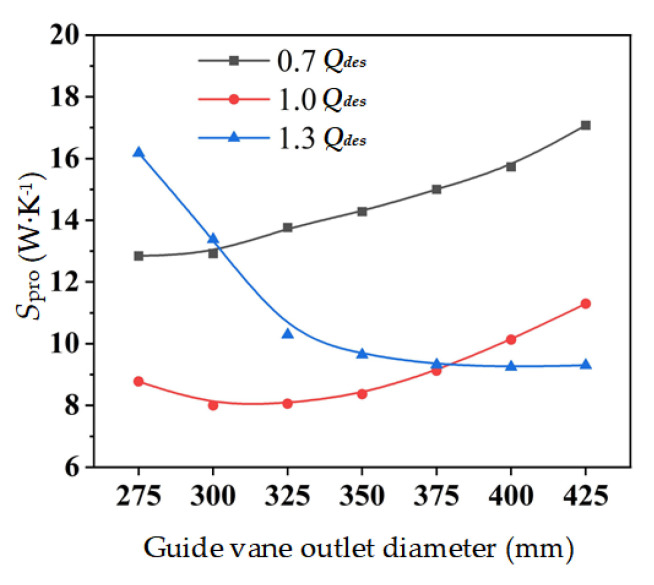
Total entropy production of the mixed-flow pump device with different guide vane outlet diameters.

The entropy production of the mixed-flow pump device with different guide vane outlet diameters under different flow conditions, is shown in Figure 9. At 0.7 *Q*_des_, the entropy production of the device increases with the increase of *D*_gvo_, and it increases by 33% as *D*_gvo_ expands from 275 mm to 425 mm. At 1.0 *Q*_des_, when *D*_gvo_ ranges from 300 to 325 mm, the entropy production is the minimum. When *D*_gvo_ < 300 mm, the entropy production raises with the decrease of *D*_gvo_, and when *D*_gvo_ > 325 mm, the entropy production raises with the increase of D. At 1.3 *Q*_des_, the entropy production of the device declines as *D*_gvo_ boosts, when *D*_gvo_ expands from 275 mm to 425 mm, it reduces by 42.5%.

**Figure 10:**
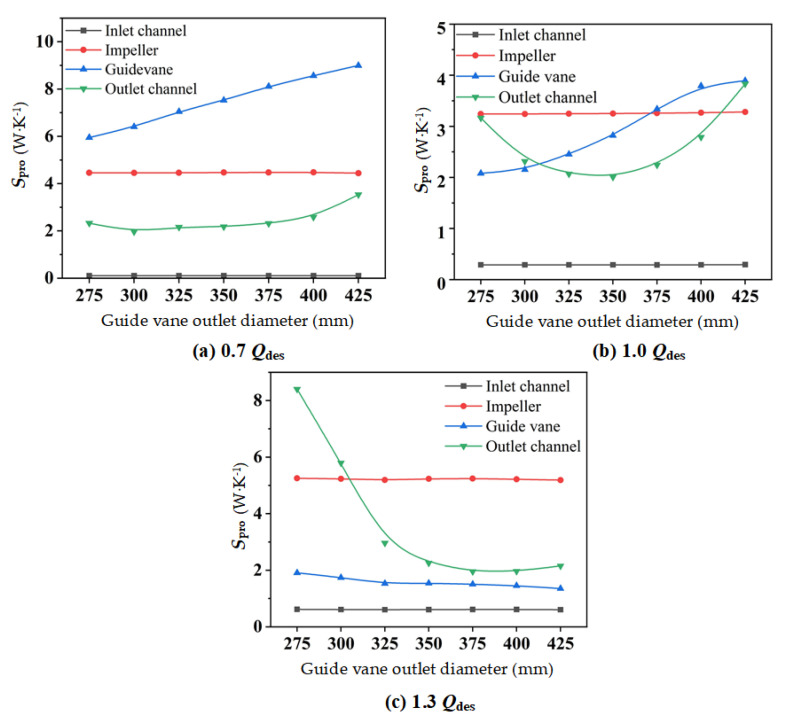
Total entropy production of each component of the mixed-flow pump device with different *D*_gvo_ at different flow rates: (**a**) 0.7 *Q*_des_; (**b**) 1.0 *Q*_des_; and (**c**) 1.3 *Q*_des_.

In Figure 10, the entropy production of each component of the mixed-flow pump device with distinct *D*_gvo_ at various flow rates, is presented. As illustrated in Figure 10, the entropy production of the inlet channel and impeller changed little under the three flow conditions, while the entropy production of the guide vane and the outlet channel showed significant changes. Therefore, the outlet channel and the guide vane were further analyzed. 

At 0.7 *Q*_des_ and 1.0 *Q*_des_, the entropy production inside of the guide vane raised as *D*_gvo_ increased, while at 1.3 *Q*_des_, there was no obvious change to entropy production. As for the outlet channel, at 0.7 *Q*_des_, with *D*_gvo_ in the range of 300 mm to 375 mm, the entropy production showed little change, but when *D*_gvo_ > 375 mm, the entropy production increased. At 1.0 *Q*_des_, when *D*_gvo_ was smaller than 350 mm, the entropy production increased as *D*_gvo_ decreased. When *D*_gvo_ was 350 mm, it reached the minimum. When *D*_gvo_ was larger than 350 mm, the entropy production increased as *D*_gvo_ increased. At 1.3 *Q_des_*, when *D_gvo_* < 325 mm, the hydraulic loss inside of the outlet increased rapidly.

### 4.4. Spread of Local Entropy Production in the Guide Vane

**Figure 11:**
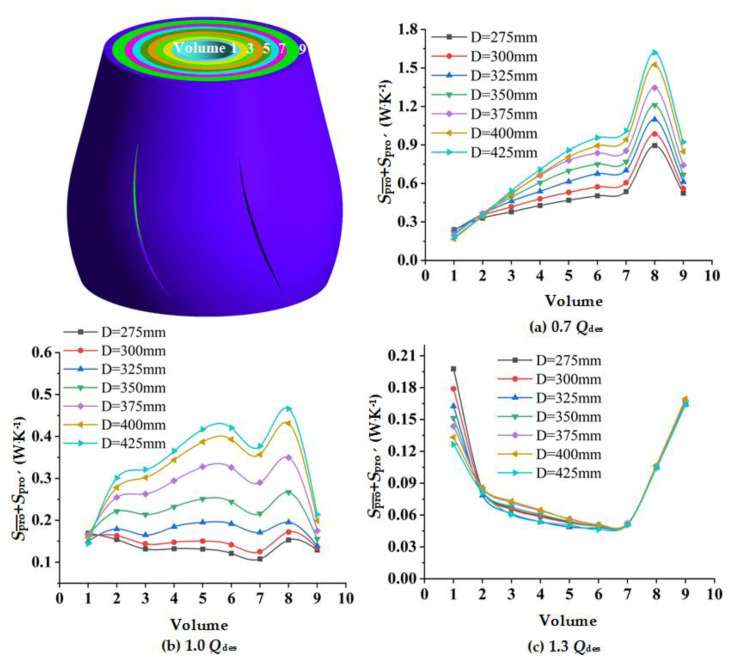
Entropy production of different volumes of guide vanes with different *D_gvo_* at various flow rates: (**a**) 0.7 *Q*_des_; (**b**) 1.0 *Q*_des;_ and (**c**) 1.3 *Q*_des_.

**Figure 12:**
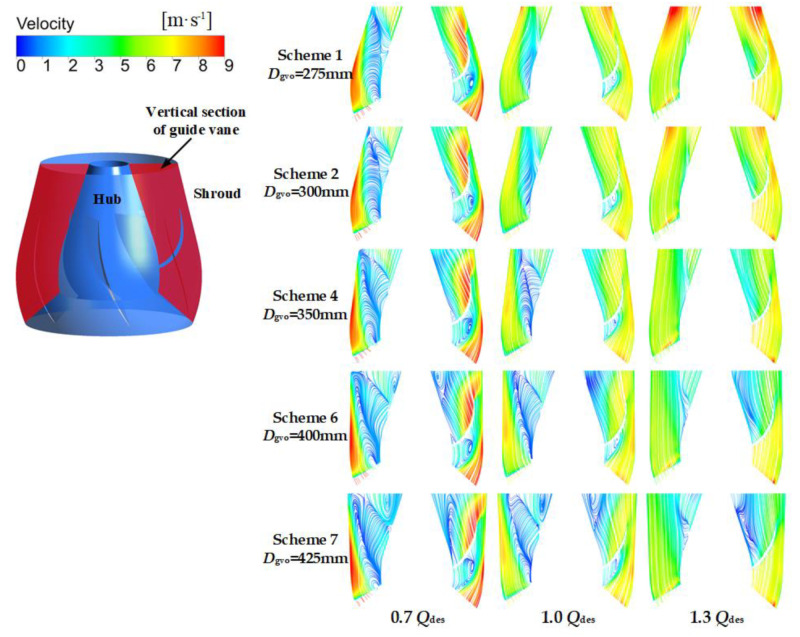
Velocity streamline of guide vanes with different meridians at various flow rate conditions.

In order to obtain the detailed distribution of entropy production in the guide vane, the guide vane was divided into nine regions from hub to shroud. The direct and indirect entropy production in each region was calculated. Since there is no wall between regions, the wall entropy production was not taken into consideration. Due to the fact that there are many guide vane schemes, only the representative Schemes 1, 2, 6, 7, and the original schemes, were selected for analysis. As shown in Figure 11, at 0.7 *Q*_des_ and 1.0 *Q*_des_, the entropy production of each region in the guide vane increased together with the increase of *D*_gvo_. According to the velocity streamline of the guide vane vertical section in Figure 12, it can be concluded that the increase of *D*_gvo_ leads to the expansion of the flow separation near the hub-side, which further leads to the increase of dissipation. It can be seen that at the small and designed flow rate conditions, reducing *D*_gvo_ is conducive to the stable flow of liquid, and thus reduces entropy production. At 1.3 *Q*_des_, the flow separation vortices on the hub side increased as *D*_gvo_ increased. However, when *D*_gvo_ is too small, the dissipation will also increase due to the excessive flow rate. Therefore, there was little difference between the entropy production of guide vanes in different schemes.

### 4.5. Spread of Local Entropy Production in the Guide Vane–Outlet Channel Interface

**Figure 13:**
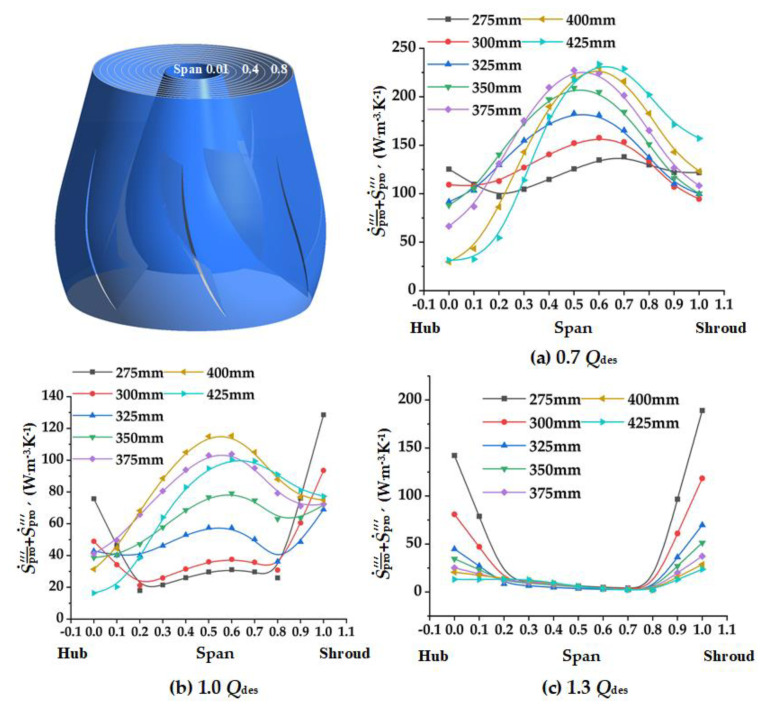
Total entropy production rate of the guide vane outlet under various flow rates: (**a**) 0.7 *Q*_des_; (**b**) 1.0 *Q*_des *;*_ and (**c**) 1.3 *Q*_des_.

**Figure 14:**
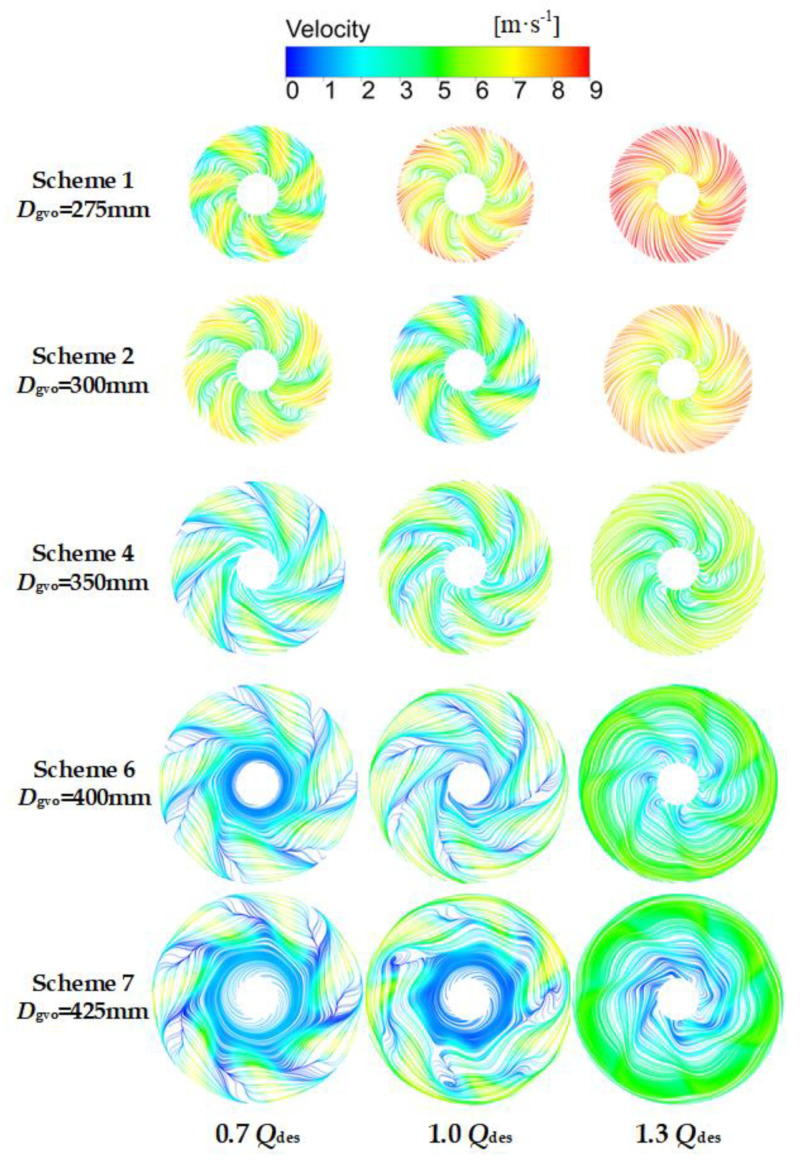
Velocity streamline of the guide vane outlet at various flow rates conditions.

There is an interference effect between the guide vane and outlet channel, so it is necessary to analyze the entropy production distribution at this interface. In this section, 11 turbo lines were constructed from hub to shroud, and the average total entropy production rate in these lines was calculated by adding direct dissipation ${\dot{S}}_{\bar{\mathrm{pro}}}^{\prime \prime \prime }$ and indirect dissipation ${\dot{S}}_{{\mathrm{pro}}^{\prime }}^{\prime \prime \prime }$ Figure 13 depicted the spread of the total entropy production rate at the guide vane–outlet channel interface. As shown in Figure 13, under all flow conditions, the total entropy production rate near the hub decreases with the increase of *D*_gvo_. According to the velocity streamline in Figure 14, this is because the increase of *D*_gvo_ enlarges the low velocity area near the hub. Under 0.7 *Q*_des_, at span 0.2–0.6, flow separation intensified with the increase of *D*_gvo_, leading to the increase of total entropy production rate, which reached a peak near span 0.6. A similar situation occurs at 1.0 *Q*_des_, but there is a significant difference. The flow separation of Scheme 7 is weaker than that of Scheme 6, so the total entropy production rate of Scheme 7 is smaller. When *D*_gvo_ < 300 mm, the total entropy production rate near the shroud decreases with the increase of flow rate, but when *D*_gvo_ > 300 mm, the opposite trend appears.

### 4.6. Spread of Local Entropy Production in Outlet Channel

**Figure 15:**
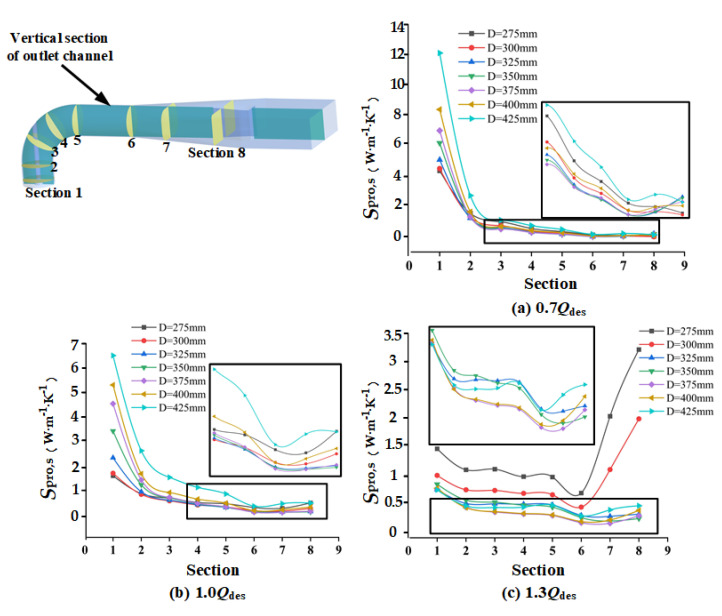
Entropy production of different sections in outlet channel under various flow rates: (**a**) 0.7 *Q*_des_; (**b**) 1.0 *Q*_des_; and (**c**) 1.3 *Q*_des_.

**Figure 16:**
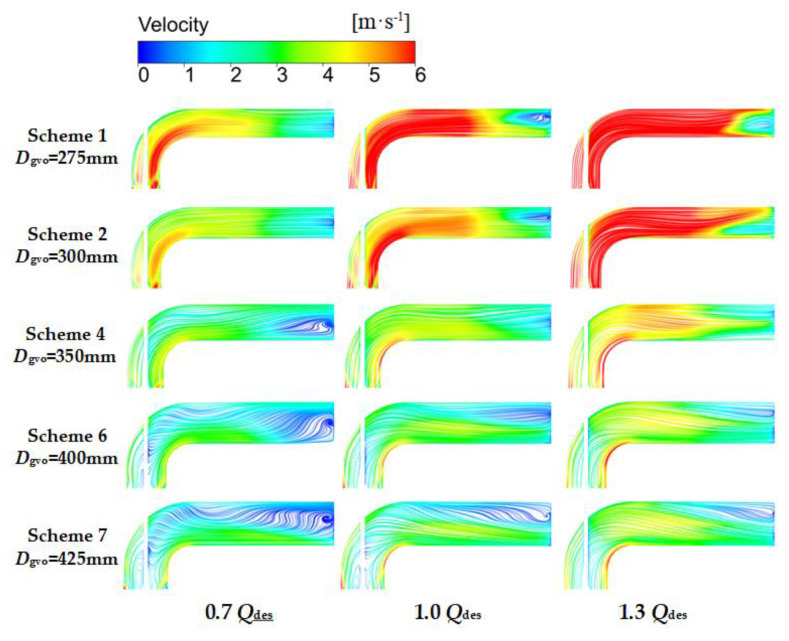
Velocity streamline of the outlet channel at various flow rate conditions.

In order to obtain the specific distribution of entropy production in the outlet channel, eight sections were established in the outlet channel, and the entropy production of these sections was calculated using Equations (11)–(13). As shown in Figure 15, at 0.7 *Q*_des_, the entropy production on the cross section decreased along the flow direction. When *D*_gvo_ = 425 mm, the entropy production was the largest, followed by *D*_gvo_ = 275 mm and *D*_gvo_ = 400 mm. Figure 16 confirms this. When *D*_gvo_ > 400 mm, a large-scale vortex appeared in the outlet channel, and when *D*_gvo_ = 275 mm, the dissipation increased due to the high velocity. At 1.0 *Q*_des_, the entropy production of *D*_gvo_ was low in the range of 325–375 mm. When *D*_gvo_ exceeded this range, the entropy production increased significantly. Combined with Figure 16, it can be seen that this was because there were vortexes in Schemes 1, 2, 6, and 7, at Sections 7 and 8, and the velocities of Schemes 1 and 2 decreased too fast at Sections 5–8, which all led to the increase of entropy production. When *D*_gvo_ = 350 mm, the flow was stable and uniform and there was no vortex. At 1.3 *Q*_des_, when *D*_gvo_ = 275 mm and 300 mm, the entropy production of each section in the outflow channel was significantly larger than that of other schemes. Especially at Sections 7 and 8, the rapid decrease of velocity of fluid led to the sharp increase of entropy production. When *D*_gvo_ was in the range of 375–400 mm, the entropy production was the smallest, the flow velocity was more uniform, and there was no obvious vortex.

(11) $$S_{\bar{\mathrm{pro},\;s}}=\int_{S}{\dot{S}}_{\mathrm{pro}}^{\prime \prime \prime }dS$$

(12) $$S_{\mathrm{pro},s^{\prime }}=\int_{S}{\dot{S}}_{{\mathrm{pro}}^{\prime }}^{\prime \prime \prime }dS$$

(13) $$S_{\mathrm{pro},s}=S_{\bar{\mathrm{pro},s}}+S_{\mathrm{pro},s^{\prime }}$$

Here, $S_{\bar{\mathrm{pro},s}}$ represents surface direct entropy production, $S_{\mathrm{pro},s^{\prime }}$ represents surface indirect entropy production, and $S_{\mathrm{pro},s}$ represents the total entropy production of the surface.

**Figure 17:**
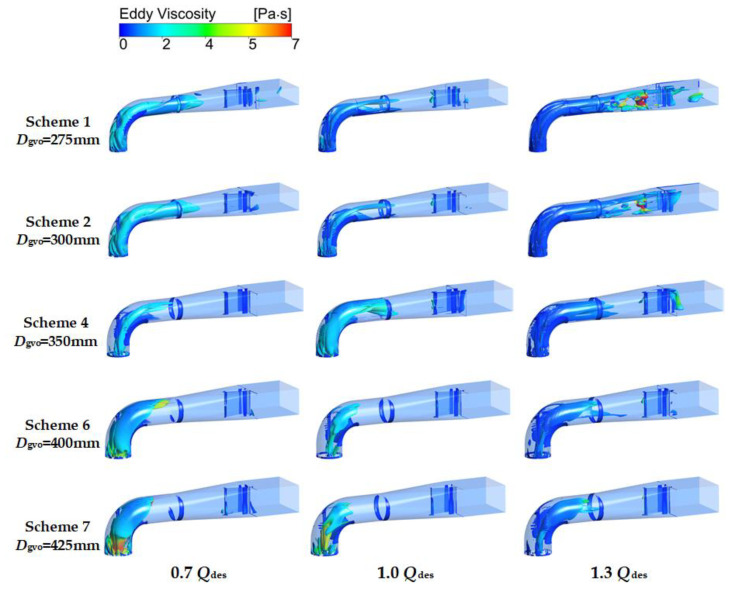
Vortex structure of the outlet channel at various flow rate conditions.

The vortex identification technology based on λ_2_ criterion [28] is used with the aim of observing the vortex structure. The study of Mariotti [29] shows that λ_2_ is a very powerful tool in the comparison between the different configurations in terms of vortical structures. According to the study of Ji [30], it is feasible to set the threshold value as 175 s^−2^ to study the vortex structure of the mixed-flow pump. As is shown in Figure 17, under all flow rate conditions, with the increase of *D*_gvo_, the vorticity of the outlet channel decrease, at the same time, the eddy viscosity augment at 0.7 *Q*_des_ and 1.0 *Q*_des_, but there is not obvious raise at 1.3 *Q*_des_. It is worth noting that when *D*_gvo_ < 300 mm, a region of high eddy viscosity appeared in the second half of the outlet channel, which resulted in the increase of entropy production. A conclusion can be drawn by comparing Figure 16 and Figure 17, when the velocity gradient of liquid is too large, vortex will be generated and entropy production will increase. Moreover, the greater the velocity gradient is, the larger the eddy viscosity is, and the higher the entropy production is.

## 5. Conclusions

In this paper, mixed-flow pump devices with different guide vane meridians were studied based on CFD and entropy production theory. By comparing CFD results with the test results of the initial scheme, the accuracy of the CFD results was verified; by comparing the hydraulic loss and entropy production of the initial scheme, the reliability of entropy production theory was verified. Based on this, the external characteristics and total entropy production of the mixed-flow pump device and the spatial distribution of the entropy production in the guide vane and outlet channel were analyzed. Conclusions can be drawn as follows:

(1) With the decrease of *D*_gvo_, the head and efficiency of the mixed-flow pump device increased at 0.7 *Q_des_*. When *D*_gvo_ decreased from 350 mm to 275 mm, the head and efficiency increased by 2.78% and 3.05%, respectively. At 1.3 *Q*_des_, when D decreased from 350 mm to 275 mm, the head and efficiency increased by 4.49% and 3.71%, respectively. Under 1.0 *Q*_des_, the head and efficiency were higher when *D*_gvo_ was in the range of 300–350 mm. Total entropy production showed an opposite trend to the head and efficiency. The entropy production of the inlet channel and impeller did not change much, but that of the guide vane and outlet channel showed significant changes.
(2) At 0.7 *Q*_des_ and 1.0 *Q*_des_, the entropy production of the guide vane increased with the increase of *D*_gvo_, which was mainly brought about by the flow separation and backflow. At 1.3 *Q*_des_, with the increase of *D*_gvo_, the flow rate of fluid in the guide vane decreased, resulting in a slight decrease in the entropy production of the guide vane.
(3) The entropy production at the interface between the guide vane and outflow channel increased with the increase of *D*_gvo_. At 0.7 *Q*_des_ and 1.0 *Q*_des_, a high entropy production area was located at the middle partial shroud of the interface, while at 1.3 *Q*_des_, it was located at the middle partial hub. The reason for the increase was the flow separation.
(4) When *D*_gvo_ decreased from 350 mm to 275 mm, the entropy production changed little at 0.7 *Q*_des_, but it soared at 1.0 *Q*_des_ and 1.3 *Q*_des_ due to excessive flow velocity. When *D*_gvo_ increased from 350 mm to 425 mm, under 0.7 *Q*_des_ and 1.0 *Q*_des_, because of the expansion of the channel section, the flow separation intensified and the entropy production increased, but the entropy production decreased slightly at 1.3 *Q*_des_.

These conclusions provide guidance for improving the operation efficiency of pumping stations. For example, when the flow rate of pumping stations is small, it is possible to reduce *D*_gvo_ to improve the efficiency, and vice versa.

## Figures and Tables

**Table 1:** Parameters of the guide vane blades.

Parameters	Value
Number of guide vane blades *Z*_2_	7
*Inlet angle α*	
Inlet angle of section A-A *α*_1_ (°)	55
Inlet angle of section B-B *α*_2_ (°)	44
Inlet angle of section C-C *α*_3_ (°)	35
*Outlet angle β*	
Outlet angle of section A-A *β*_1_ (°)	90
Outlet angle of section B-B *β*_2_ (°)	90
Outlet angle of section C-C *β*_3_ (°)	90
*Wrap angle φ*	
Wrap angle of section A-A *φ*_1_ (°)	21.2
Wrap angle of section B-B *φ*_2_ (°)	33.6
Wrap angle of section C-C *φ*_3_ (°)	55.3
*Length of guide vane blades*	
Length of guide vane at section A-A *L_A_* (mm)	229
Length of guide vane at section B-B *L_B_* (mm)	205
Length of guide vane at section C-C *L_C_* (mm)	180

**Table 2:** Head and efficiency under different grid numbers.

Grid Number/× 10^6^	Head/m	Efficiency/%
2.06	8.002	81.12
3.52	8.097	81.48
4.98	8.085	81.34
6.57	8.105	81.27
8.54	8.102	81.39

**Table 3:** Details of boundary conditions.

Boundary Conditions	Boundary Type
Inlet of pump device	Mass flow rate
Outlet of pump device	Opening
Solid wall	No-slip
*Interface on both sides of the impeller*	
Transient state	Transient rotor-stator
Steady state	Frozen rotor

## Data Availability

Not applicable.

## Nomenclature

*D*_gvo_	guide vane outlet diameter (mm)
*Q*_des_	design flow rate (L/s)
*L*	length of guide vane blades (mm)
*Z*	number of blades
*E*_Q_	uncertainty of flow measurement (%)
*E*_H_	uncertainty of head measurement (%)
*E*_M_	uncertainty of torque speed measurement (%)
*E*_N_	uncertainty of rotating speed measurement (%)
*E*_S_	uncertainty of the test bench (%)
*E*_R_	uncertainty of efficiency measurement (%)
*n*	rotation speed of the impeller (r/min)
$\bar{u}$	averaged velocity (m·s^−1^)
$u^{\prime }$	fluctuating velocity (m·s^−1^)
${\dot{S}}_{}^{}$	entropy production rate (W·K^−1^·m^−3^)
*T*	temperature (K)
*k*	turbulent eddy dissipation
$S_{}$	entropy production (W·K^−1^)
Greek symbols	
μ	fluid viscosity (kg·m^−1^·s^−1^)
*α*	inlet angle of guide vane blades (°)
*β*	outlet angle of guide vane blades (°)
*φ*	warp angle of guide vane blades (°)
